# Sustainable Lignin-Reinforced Chitosan Membranes for Efficient Cr(VI) Water Remediation

**DOI:** 10.3390/polym16131766

**Published:** 2024-06-21

**Authors:** Ana S. Castro, Bárbara D. D. Cruz, Daniela M. Correia, Senentxu Lanceros-Méndez, Pedro M. Martins

**Affiliations:** 1 Centre of Chemistry, University of Minho, 4710-057 Braga, Portugal; 2 Institute of Science and Innovation on Bio-Sustainability (IB-S), University of Minho, 4710-057 Braga, Portugal; 3 Centre of Molecular and Environmental Biology, University of Minho, 4710-057 Braga, Portugal; 4 Physics Centre of Minho and Porto Universities (CF-UM-UP), Laboratory of Physics for Materials and Emergent Technologies, LapMET, University of Minho, 4710-057 Braga, Portugal; 5 BCMaterials, Basque Center for Materials, Applications and Nanostructures, UPV/EHU Science Park, 48940 Leioa, Spain; 6 Ikerbasque, Basque Foundation for Science, 48009 Bilbao, Spain

**Keywords:** adsorption, chromium, membranes, environmental remediation, chitosan/lignin, sustainable biopolymers

## Abstract

The pollution of aquatic environments is a growing problem linked to population growth and intense anthropogenic activities. Because of their potential impact on human health and the environment, special attention is paid to contaminants of emerging concern, namely heavy metals. Thus, this work proposes the use of naturally derived materials capable of adsorbing chromium (VI) (Cr(VI)), a contaminant known for its potential toxicity and carcinogenic effects, providing a sustainable alternative for water remediation. For this purpose, membranes based on chitosan (CS) and chitosan/Kraft lignin (CS/KL) with different percentages of lignin (0.01 and 0.05 g) were developed using the solvent casting technique. The introduction of lignin imparts mechanical strength and reduces swelling in pristine chitosan. The CS and CS/0.01 KL membranes performed excellently, removing Cr(VI) at an initial 5 mg/L concentration. After 5 h of contact time, they showed about 100% removal. The adsorption process was analyzed using the pseudo-first-order model, and the interaction between the polymer matrix and the contaminant was attributed to electrostatic interactions. Therefore, CS and CS/KL membranes could be low-cost and efficient adsorbents for heavy metals in wastewater treatment applications.

## 1. Introduction

Fresh water represents approximately 3% of the total water available on planet Earth, but only a small percentage, ≈0.01%, of fresh water is available for consumption [1]. The quality of this water directly affects human health and well-being [2]. In recent decades, water pollution has been associated with population growth and multiple, increasingly intense human activities, such as industry, transport, agriculture, and urbanization [3]. An important concern in this context is the rise of so-called emerging pollutants, representing an increased problem because they are persistent, are present in the environment at low concentrations (ng/L to μg/L), and are potentially bioaccumulative and toxic [3,4,5].

Heavy metal ions in industrial wastewater seriously threaten the lives of plants, animals, microorganisms, and humans [6]. These non-biodegradable contaminants enter the food chain, leading to bioaccumulation and toxicity to living organisms [7,8,9]. Recognized by the World Health Organization (WHO) as one of the top 10 chemical pollutants, Cr(VI) has seen an alarming increase in its presence in water bodies [10,11,12,13]. Industries such as electroplating, tanneries, mines, pigments, cement, paints, steel, metallurgy, textiles, and the manufacture of alloys contribute significantly to the release of chromium into the environment [14]. It exists in two stable oxidation states, Cr (III) and Cr(VI) [14,15]. Cr (III) is an essential trace element for human metabolism, whereas Cr(VI) is potentially toxic and carcinogenic [16]. The WHO defined an acceptable limit of 0.05 ppm for chromium in water [17]. Exceeding these standards can cause serious health problems, including allergies, skin ulcers, liver and kidney damage, headaches, nausea, diarrhoea, and vomiting [18]. Because of these risks, there is an urgent need to remove Cr(VI) from water sources. 

Nowadays, there are a variety of techniques that can effectively remove heavy metals from aqueous media, such as chemical precipitation [19], ion exchange [20], membrane filtration [21], coagulation [22], flocculation [23], reverse osmosis [24], biological processes [25], and adsorption [26]. The selection of techniques to remove one or several types of heavy metals is generally based on costs, efficiency, reliability, feasibility, environmental impact, practicality, and difficulties in application [27]. Given these requirements, adsorption has received considerable attention due to the advantages of the process over alternative approaches. The adsorption method is simple and easy to operate, non-toxic, capable of removing various contaminants, highly selective, and applicable over a wide pH range [19,28,29]. The limitation of this process is the loss of adsorption capacity in regeneration cycles and the need for the chemical regeneration of the adsorbent [29]. 

Adsorption is a mass transfer process involving atoms, ions, or molecules from a liquid to the solid surface of a substance via physical or chemical interactions [29]. Several studies have been reported using different types of adsorbents to remove Cr(VI) from water, ranging from membranes based on metal particles [30], minerals [31], polymers [32], carbon-based materials [33], and biological materials [34]. However, in recent decades, natural-based polymers have attracted more interest due to their easy processability [35], low environmental impact, low cost, efficiency, low need for chemicals, and regeneration and recycling ability, promoting the development of more sustainable solutions for water contamination [36,37].

Chitosan (CS) is a natural biopolymer derived from the deacetylation of chitin, a polysaccharide obtained from the beaks of cephalopods, the cell walls of fungi, and the exoskeletons of shellfish and crustaceans [38]. This biopolymer has gained prominence in wastewater treatment due to its favorable aspects, including its low cost, abundance, non-toxicity, good adsorption capacity, and selectivity for metal ions such as Cr(VI) [39]. It also has antimicrobial and antioxidant properties, as well as biodegradable, biocompatible, and sustainable attributes [40,41,42]. This biopolymer has already been featured in several studies in the removal of heavy metals, as it has hydroxyl (-OH) and amine (-NH_2_) groups, which bond bridges with the contaminant, giving it a good adsorption capacity [43]. Despite its remarkable adsorption capacity, chitosan has drawbacks such as solubility in water at acidic pH and high shrinkage after drying [44]. To overcome these limitations, it is modified with complementary materials to form composites or blends, making it possible to improve some of the properties of CS. There is a wide variety of chitosan-based composites capable of adsorbing chromium, including the addition of nanoparticles like ZnO [45], graphene oxides [46], and carbon nanotubes [47], or the inclusion of metal–organic frameworks (MOFs) [48] and ionic liquids [49] as fillers. This allows for improved mechanics, tensile strength, and greater permeability and selectivity [50,51]. Another alternative is to add other polymers to CS, including poly(vinylidene fluoride) (PVDF) [52], polyethersulfone (PES) [53], alginate [54], cellulose [55], or lignin [56], among others.

Lignin is a natural biopolymer with an amorphous, three-dimensional, aromatic structure [57]. It is a residual by-product of the pulp and paper industry and can vary in composition and structure depending on the extraction process [58]. Kraft lignin (KL) has a substantially higher adsorption affinity due to breaking aryl ether bonds during the Kraft pulping process [59]. This biopolymer has several advantages, such as its availability, low cost, biodegradability, antioxidant properties, insolubility in water, high thermal stability, and rigidity [59,60]. Further, it shows adsorption capabilities due to the polymer’s active sites (hydroxyl, carboxyl, methoxide, and aldehyde groups) that are suitable for heavy metal removal [61]. In this way, lignin and chitosan share some characteristics, such as viable accessibility, biocompatibility, and environmentally beneficial properties. In addition, they are a good alternative to polymers made from fossil resources, and several studies prove their affinity for adsorbing heavy metals [29,56].

Despite its objective interest and potential, the application of membranes based on chitosan and lignin in environmental remediation has yet to be explored. In this context, membranes based on chitosan and lignin were developed, with different concentrations of lignin (0.01 and 0.05 g), and their applicability in Cr(VI) removal was demonstrated.

## 2. Experimental

### 2.1. Materials

Low-molecular-weight chitosan powder with a degree of deacetylation of ≥75% was obtained from Sigma-Aldrich (CAS: 9012-76-4), St. Louis, MO, USA. Lignin processed by Kraft was provided by Sigma-Aldrich (CAS: 8068-05-1), St. Louis, MO, USA. Acid acetic, ≥99.5% (CH_3_COOH), was purchased from Fisher Chemical, Hampton, NH, USA. Potassium dichromate (K_2_Cr_2_O_7_) and sulphuric acid (H_2_SO_4_) were acquired from Merck, Darmstadt, Germany. 1,5-Diphenylcarbazide (C_13_H_14_N_4_O) was purchased from Sigma-Aldrich, St. Louis, MO, USA.

### 2.2. Preparation of CS/KL Membranes

A simple approach was used to develop the adsorbent membranes composed of CS/KL. Three membranes were prepared via solvent casting: one pure CS and two other CS membranes containing 0.01 g and 0.05 g of lignin. Polymer concentration was maintained for the three membranes, dissolving 0.027 g in an acetic acid solution containing 0.5% *w*/*v* in deionized water. Other membranes were produced, but only those that were homogeneous and mechanically stable were produced.

**Figure 1:**
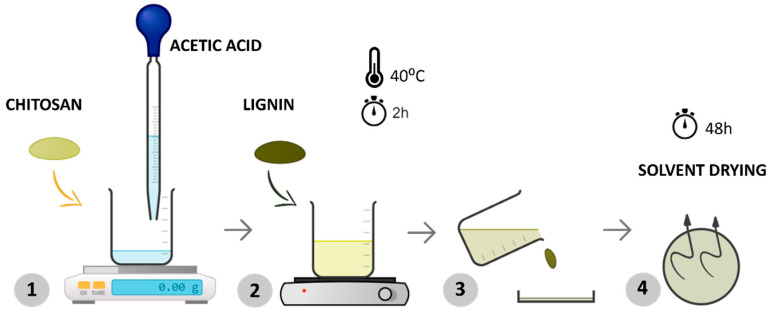
Schematic representation of the experimental procedure for processing samples via solvent casting.

In the first step, chitosan and lignin were dissolved in a 0.5% *w*/*v* acetic acid solution by magnetically stirring at 40 °C for about 2 h until the polymer was dissolved entirely (Figure 1—steps 1 and 2). Afterward, the solution was poured into a Petri dish (step 3), ensuring uniform distribution of the mixture, and the solvent was allowed to evaporate at room temperature for 48 h (step 4). This procedure is shown in Figure 1. 

After this process, membranes with a radius of 8.5 cm and an average thickness between 57 and 60 μm were obtained.

### 2.3. Characterization of CS/KL Membranes

The morphology of the CS and CS/KL composites was evaluated using a Hitachi S-4800 SEN Scanning Electron Microscope (SEM) (Tokyo, Japan) with an acceleration voltage of 20 kV. Before analysis, the samples were coated with a thin layer of gold using sputter coating (Polaron SC502, LADD Research Industries, Essex, MA, USA). The samples were analyzed cross-sectionally (prepared using liquid nitrogen) and on the surface before and after Cr(VI) adsorption. 

The attenuated total reflection (ATR) Fourier transform infrared (FTIR) spectra were obtained by placing the produced samples (CS and CS/KL) directly in a Perkin Elmer spectrometer (Waltham, MA, USA) at room temperature in the 4000 and 400 cm^−1^ range, using 16 scans and a resolution of 4 cm^−1^. The FTIR-ATR analysis of the samples did not require any previous treatment.

X-ray Diffraction (XDR) patterns were recorded with a Philips X’Pert MPD Powder detector (Eindhoven, The Netherlands), in the 2q range of 10° to 40° at room temperature. Monochromated Cu Kα radiation (λ = 1.541 Å) was used with a resolution of 0.02°. Any necessary thermal pre-treatment was undertaken prior to analysis. 

Differential Scanning Calorimetry (DSC) thermograms were recorded in a Mettler Toledo 822e calorimeter (Columbus, OH, USA) between 25 and 200 °C at a heating rate of 10 °C/min in a high nitrogen atmosphere at a constant flow of 20 mL/min. 

The mechanical properties were evaluated in the tensile mode, in triplicate for each sample and at room temperature, in a Linkam Scientific Instruments TST 360 (Surrey, UK) testing machine at a deformation speed of 15 μm/s. Samples of 30 mm × 10 mm were cut for all the experiments.

The membranes CS and CS/KL were immersed in distilled water at room temperature until the swelling time curve reached a plateau. The water uptake was calculated according to Equation (1) [62]:

(1) $$\mathrm{Water}\;\mathrm{uptake}\left(\%\right)=\frac{m_{W}-m_{d}}{m_{d}}\times 100$$

m_W_ and m*_d_* were the membrane’s wet and dry weights, respectively.

### 2.4. Adsorption Experiments of Cr(VI)

Cr(VI) adsorption tests were carried out to evaluate the adsorption efficiency and capacity over the exposure time and vary the solution pH and initial Cr(VI) concentration to assess the effect of those relevant parameters on the adsorption performance of CS and CS/KL membranes. At the beginning of the experiment, a Cr(VI) stock solution (5 mg/L) was prepared by dissolving potassium dichromate (K_2_Cr_2_O_7_) in deionized water. All the experiments used 50 mL of Cr(VI) solution and were performed with the same exposure time of 300 min (5 h). Further, all membranes were cut into equal sections (2 cm × 2 cm). The tests were conducted at room temperature and under magnetic stirring at 150 rpm.

The quantitative analysis of Cr(VI) was performed using the diphenylcarbazide (DPC) method according to [63,64]. DPC does not form a complex directly with Cr(VI) but is oxidized to 1,5-diphenylcarbazone (DPCO), whereas Cr(VI) is reduced to Cr(III). The reduced Cr(III) then forms a complex with DPCO. This two-step process is described as follows (Equations (2) and (3)) [63].

2Cr(VI) + 3DPC ↔ 2Cr(III) + 3DPCO + 6H^+^ (2)

Cr(III) + DPCO ↔ [CrDPCO]^+^ + 2H^+^ (3)

This method consists of taking 2.5 mL of sample and adding 2.5 mL of ultrapure water. Then, 100 μL of a previously prepared 1,5-diphenylcarbazide solution (25 mg of 1,5-diphenylcarbazide in 5 mL of acetone) was added. Further, 10 μL of sulfuric acid (H_2_SO_4_) solution (10% (*V*/*V*)) area was also added to the solution, which was then homogenized and subjected to UV-visible spectroscopy (Tecan Infinite M Nano+ Männedorf, Switzerland) evaluation at a $\lambda_{\max}$ = 540 nm [65].

The Cr(VI) removal efficiency (*E*, %) and adsorption capacity (*Qe*, mg/g) were evaluated according to Equations (4) and (5) [66]:

(4) $$E(\%)=\frac{C_{i}-C_{f}}{C_{i}}\times 100$$

(5) $$Q_{e}=\frac{{(C}_{i}-C_{f})\;}{m}\times V$$

where C*_i_* and C*_f_* represent the initial and final concentrations of solute (mg/L), respectively, and *m* and *V* represent the mass of the adsorbent (g) and the volume of the solution (l), respectively.

All of the parameters except pH were kept constant to evaluate the effect of pH on Cr(VI) removal efficiency. For this purpose, three Cr(VI) solutions were prepared at a concentration of 5 mg/L with acidic, neutral, and alkaline pHs—namely 3, 7, and 10—using a 1 M HCl solution and a 1 M NaOH solution to obtain the desired pH. Adsorption tests were carried out using CS and CS/0.01 KL membranes.

The adsorption of Cr(VI) by varying the concentration of chromium ions in solution was also evaluated with 5, 25, and 50 mg/L solutions, keeping all the other parameters constant. These tests were carried out with both CS and CS/0.01 KL membranes. The solution was removed after 5 h and analyzed using a UV-Vis spectrophotometer (Tecan Infinite M Nano+). 

In studying the Cr(VI) adsorption mechanism, the analysis of adsorption kinetics plays an essential role, allowing for an understanding of the physical and chemical interactions that govern the adsorption process. The kinetic behavior of adsorption on CS and CS/0.01 KL membranes was studied using non-linear pseudo-first order [60] (Equation 6) and pseudo-second-order [67] (Equation (7)) models:

(6) $$\ln(q_{e}-q_{t})=\ln q_{e}-K_{1}t$$

(7) $$\frac{t}{q_{t}}=\frac{1}{k_{2}{q_{e}}^{2}}+\frac{t}{q_{e}}$$

where *Qe* (mg/g) and *q_t_* (mg/g) are the adsorption capacities at a given time *t* (min) and equilibrium, respectively. *k*_1_ (min^−1^) is the first-order rate constant, and *k*_2_ (g/mg/min) is the second-order rate constant.

To confirm the presence of Cr(VI) on the surface of CS and CS/KL membranes, they were analyzed using SEM-EDX with an INCA 350 spectrometer (Oxford Instruments NanoAnalysis & Asylum Research, High Wycombe, UK). The same membranes were then analyzed via FTIR-ATR, using the previously described conditions, before and after contact with the contaminant.

## 3. Results and Discussion

### Characterization of CS/KL Membranes

**Figure 2:**
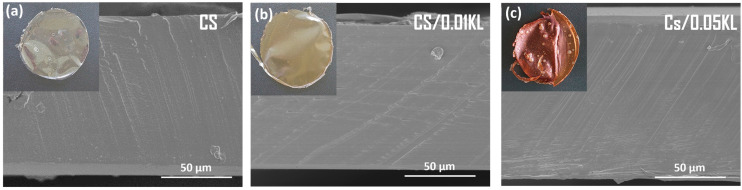
Cross-sectional SEM images of (**a**) CS, (**b**) CS/0.01 KL, and (**c**) Cs/0.05 KL.

The morphology of CS and CS composite membranes was evaluated using the SEM technique. Figure 2 shows the cross-sectional SEM images of the neat CS and CS/KL with different lignin contents. The top left corner depicts the visual aspect of each membrane with a diameter of approximately 8.5 cm. 

The cross-sectional SEM images of CS membranes exhibited a compact, non-porous texture (Figure 2a). Incorporating lignin into the CS (Figure 2b,c) matrix does not induce modifications in the microstructure of membranes. Still, in the visual aspect of the membranes, it is possible to observe a brown-colored membrane due to the addition of lignin, increasing the intensity of the color membrane with increasing lignin concentration [68].

**Figure 3:**
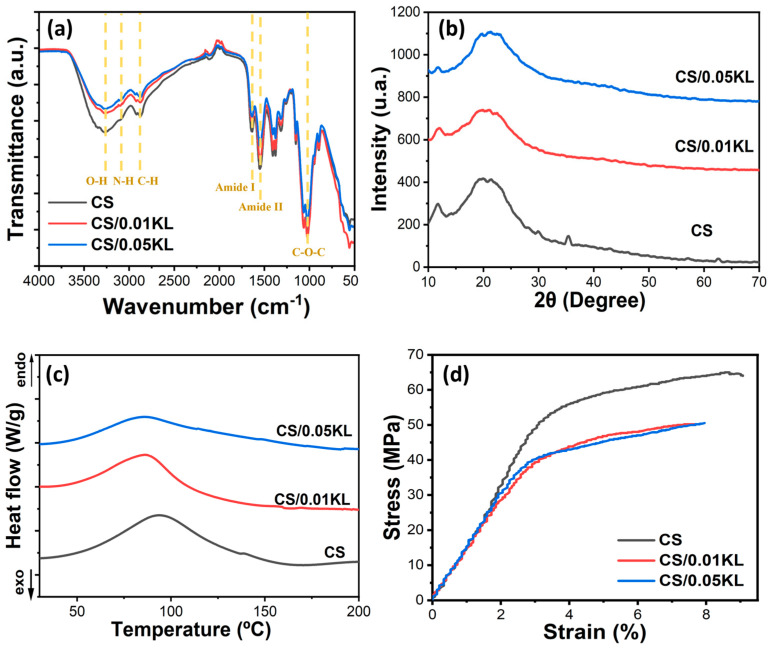
(**a**) FTIR-ATR spectra, (**b**) XRD patterns, (**c**) DSC thermograms, and (**d**) stress–strain curves of chitosan and chitosan/kraft lignin membranes at different lignin concentrations (0.01 and 0.05 g).

The FTIR-ATR analysis allowed us to analyze the potential interactions between CS and KL in the composite membranes, as shown in Figure 3a. The FTIR-ATR spectrum of the neat CS membrane is characterized by a band between 3500 and 3000 cm^−1^, corresponding to the stretching vibrations of O-H and the symmetric and asymmetric stretching of N-H bonds in the amino group of the chitosan molecules [69]. The absorption bands at 1640 cm^−1^ and 1550 cm^−1^ are associated with the vibrational modes by stretching of the C=O group and scissoring vibration of the -NH_2_ group, respectively [70]. The peaks at 1400 cm^−1^ and 1380 cm^−1^ are associated with the vibrational modes of the symmetric deformation of the CH_2_ group, and the one at 1320 cm^−1^ corresponds to the amide III group due to the deformation combination of the N-H group and C-N stretching vibration. The C-O bond is identified by the band at 1022 cm^−1^ [71]. 

According to the literature, lignin shows characteristic absorption bands between 1500 and 1420 cm^−1^ corresponding to aromatic ring vibrations and one between 3500 and 3000 cm^−1^ attributed to aromatic and aliphatic O-H groups. It also presents a band at 1021 cm^−1^ attributed to the stretching of the C-O-C bond, an absorption band at 2930 cm^−1^ corresponding to the stretching of the C-H bond of the methyl groups, and a band at 1737 cm-1 due to the stretching of the aromatic C=O bond [72]. Incorporating lignin in the chitosan matrix does not significantly change the FTIR-ATR spectra of chitosan since there are no new absorption bands or energy shifts of the existing bands, showing no chemical interactions between chitosan and lignin occurred. 

Confirming the degree of deacetylation (DD) and molecular weight claimed by the manufacturer is relevant to providing further data about CS characterization. Despite not performing NMR in this study, previous studies by the authors performed on the same commercial CS after processing have estimated a DD of 78%, which is very near to the 75% claimed by the manufacturer, confirming CS stability [73]. Furthermore, the molecular weight of the same commercial CS is between 50,000 and 190,000 Da in other works [74,75].

The possible modifications in the crystalline conformation of chitosan with the incorporation of lignin in the CS matrix were evaluated using XRD analysis. Related to the semi-crystalline characteristics of CS, Figure 3b presents two diffraction peaks at 12° and 20° that correspond to the (020) and (110) diffraction planes, respectively [76]. The diffraction peak at 2θ = 35° corresponds to a chemical substance related to the mineral component of bones and hard tissues in animals [77]. With the addition of lignin, the diffraction peaks at 12° and 20° do not change. However, there was a reduction in intensity, which could be attributed to some reduction in the crystallinity of CS [78]. 

A DSC analysis was performed to evaluate the thermal transitions of the CS matrix with the incorporation of different lignin contents (Figure 3c). The endothermic peak at 94 °C, present in the DSC thermogram of all samples, is due to the loss of water contained in the polymer backbone, which is bonded to the structure of CS of the in-substituted free -OH and -NH_2_ groups [78]. The incorporation of lignin causes a slight shift toward low temperatures in the endothermic peak, indicating that its addition does not lead to relevant variations in the thermal characteristics of the CS polymer matrix.

Stress–strain tests were carried out to evaluate the effect of adding lignin in the CS matrix on the mechanical properties of the membranes. Figure 3d presents the results of the tensile stress measurements acquired for the CS and CS/KL composite membranes with different KL contents. The tensile strength (σy), elongation at break (εb), and Young’s Modulus (E) are shown in Table 1. 

As shown in Table 1, the pure CS membrane is characterized by a tensile strength of 55.58 ± 8.12 MPa and an elongation at a break of 7.04 ± 2.02%. With the incorporation of 0.01 and 0.05 g lignin, the tensile strength decreases slightly, and the elongation at the break slightly increases. This increase may be caused by the interaction between lignin and chitosan, which promotes better adhesion between the molecules of the two polymers and results in greater elongation at break, as seen in Table 1 [62,79]. These results are aligned with the literature, which mentions that adding different percentages of lignin does not affect the mechanical properties of the membranes [80]. Regarding the Young’s Modulus, calculated at 3% of deformation, it slightly decreases in the lignin-chitosan blend membranes from 15.95 ± 0.92 MPa to neat CS to 13.49 ± 1.13 MPa from the membrane of CS/0.05 kL, indicating that the addition of lignin induces a slight plasticizing effect into the membrane. 

**Figure 4:**
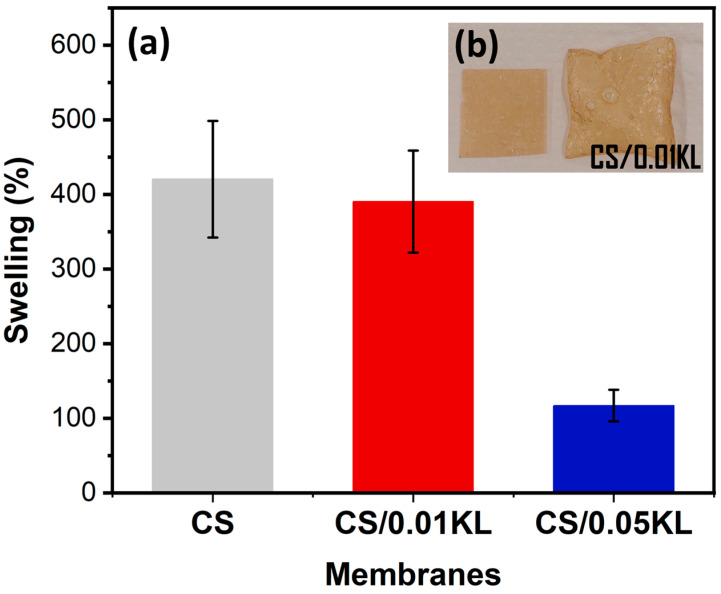
Water swelling results for CS and CS/KL membranes in water after 300 min (**a**) and the visual result of the swelling of a CS/0.01 KL membrane (**b**).

Swelling measurements were carried out using water and at room temperature for the CS and CS/KL membranes. Figure 4a shows the swelling results in percentages, and Figure 4b shows a CS/0.01 KL membrane before and after swelling for 300 min. 

The CS sample shows a degree of water swelling of 420% after 300 min. For the membranes with different amounts of lignin, the membrane with the lowest amount (CS/0.01 KL) developed a swelling of 391%, and the sample CS/0.05 KL had a swelling value of 117%. 

According to [62,80], the results indicate that swelling decreases as the lignin concentration increases. The observed swelling decrease can result from the interactions between the chitosan and lignin molecules, which induce good compatibility between the two components and reduce water adsorption [62].

## 4. Adsorption of CS/KL Membranes

### 4.1. Effect of pH

**Figure 5:**
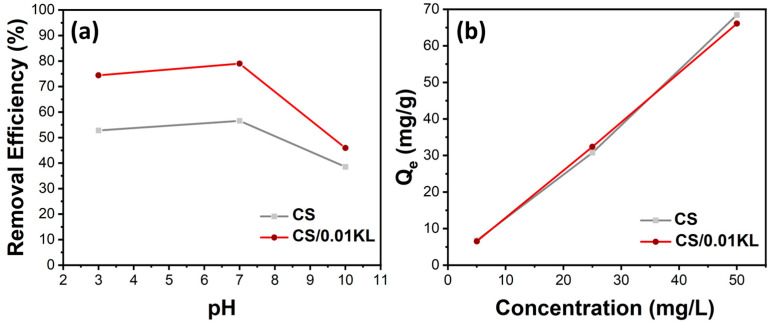
Effect of (**a**) pH variation on Cr(VI) removal efficiency (%) C_i_[Cr(VI)] = 5 mg/L) and (**b**) initial concentration of C_i_[Cr(VI)] = 5, 25, and 50 mg/L on Qe (mg/g) (stirring speed 150 rpm, contact time 5 h, room temperature).

During the Cr(VI) adsorption tests, some parameters were varied, which could influence the adsorption efficiency/capacity of the membranes. Considering the SEM and mechanical results, the sample containing 0.05 g of lignin displays a heterogeneous dispersion with similar mechanical properties. In this way, we selected the pure CS and the CS/0.01 KL to perform all the adsorption assays. Initially, the effect of pH on Cr(VI) adsorption was studied, as it determines the degree of electrostatic or molecular interaction between the surface of the adsorbent and the adsorbate due to the distribution of charges in the material [56,81]. Therefore, Cr(VI) adsorption by CS and CS/0.01 KL membranes was tested at different pH values ranging from acidic to basic solutions, i.e., 3, 7, and 10 pH, as illustrated in Figure 5a).

**Figure 6:**
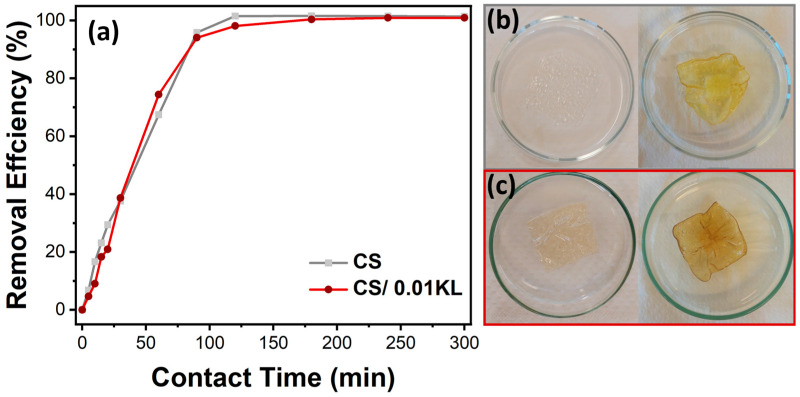
(**a**) Effect of contact time on the adsorption of chromium ions on CS and CS/0.01 L membranes (Cr(VI) concentration = 5 mg/L, stirring speed = 150 rpm, room temperature). (**b**) Visual representation of the CS and (**c**) CS/0.01 L membranes before (left) and after 300 min (suitable) of contact with Cr(VI).

The relationship between the initial pH of the solution and the percentage of Cr(VI) removal efficiency is shown in Figure 6a. The percentage removal of Cr(VI) for the CS membrane at pH 3 is 53%; at a neutral pH, it is 57%; and at an alkaline pH, the efficiency is 38%. The literature shows that the highest removal efficiencies occur at acidic pH [82,83]. Since at lower pHs, the concentration of H+ ions is higher, and since the functional groups of chitosan, -OH, and -NH, are highly protonated (positively charged), these groups are highly favorable for the adsorption of Cr(VI) anionic ions by electrostatic attraction forces. Regarding the CS/KL membrane, the Cr(VI) removal at different pHs attained higher values for all the tested pHs of 74, 79, and 46% for pH 3, 7, and 10, respectively. These results indicate that lignin is vital in the Cr(VI) removal process. Other works have studied the role of lignin in Cr(VI) adsorption; for instance, Demirbas indicates that the adsorption of Cr(VI) on modified lignin is mainly due to the electrostatic attraction between the Cr(VI) ions and the protonated carboxyl groups present on the surface of the lignin at a low solution pH. When the pH of the solution decreases, H^+^ associates with functional groups such as carboxylic, phenolic, hydroxyl, and carbonyl groups, thus increasing the affinity for Cr(VI) ions because they are positively charged (protonated) [84]. On the other hand, the lower adsorption of Cr(VI) at a high pH is perhaps due to the occurrence of an excess of -OH ions that compete with the negatively charged Cr(VI) ions for the exchange sites on the biosorbent [85]. Such findings are aligned with our results, as higher efficiencies were obtained for samples with lignin and even higher for acidic and neutral pH’s.

The effect of the initial concentration of Cr(VI) on adsorption capacity (Qe) is shown in Figure 5b. The adsorption capacity of the CS and CS/0.01 KL membranes was evaluated by placing them in contact with different Cr (VI) initial concentrations, namely 5, 25, and 50 mg/L. The results indicate that both membranes’ adsorption capacity increased as the Cr (VI) initial concentration increased, indicating that the membrane’s active sites are not saturated with the range of concentrations tested, from 5 to 50 mg/L [82,86]. Furthermore, despite the swelling and mechanical changes previously indicated and discussed after signing incorporation into the CS matrix, the adsorption capacity of CS/L membranes was not significantly improved with the presence of lignin.

### 4.2. Effect Contact Time

The study of Cr(VI) adsorption as a function of contact time allows for the evaluation of the kinetics of the adsorption process. The adsorption efficiency of Cr(VI) at an initial concentration of 5 mg/L by the CS and CS/0.01 L membranes over 5 h (300 min) was thus analyzed, as shown in Figure 6a. 

It is observed that the removal efficiency over time is similar for both membranes. The abundance of unoccupied active sites in the CS membrane causes an accelerated removal rate in the first 60 min of contact. After this period, efficiencies of about 67% and 74% are observed for the CS and CS/0.01 KL membranes, respectively. Subsequently, there was a gradual reduction in the adsorption rate until 120 min due to decreased active sites available on the membranes. After 120 min, equilibrium was reached with approximately 100% Cr(VI) removal efficiency for both membranes. This efficiency percentage was maintained until the end of the test (300 min). Vaishakh et al. [56], in a similar study, demonstrated that the composite of chitosan/alkaline lignin (50:50 wt.%) significantly increased the Cr(VI) removal rate after 6 h of contact compared to pristine polymers.

Figure 6b,c show that after 5 h of contact with Cr(VI), the CS membrane became significantly yellow, and the CS/0.01 KL membrane became slightly brown, indicating a visual difference after contact with the Cr(VI) solution.

Table 2 compares the Cr(VI) adsorption efficiency obtained in the present study with related chitosan-based membranes from the literature. 

The table outlines the removal efficiency of Cr(VI) in percentage (%) based on the initial concentration and the contact time with the contaminant. In all the results analyzed, the removal of the contaminant is greater than 50%, achieved within a contact time of up to 420 min (7 h). A study by Sheth et al. [80] used a smaller amount of adsorbent to remove a higher concentration of Cr(VI) than the Samuel et al. study [44]. In addition, it manages to remove it in less contact time (360 min). However, the removal efficiency was lower in the Sheth et al. study [80].

A study by Saini et al. [87] is similar to this study, as it has a contact time and initial concentration closer to those tested. In this study, various compounds were tested, including chitosan/lignin, which achieved a removal efficiency of 91.69%. Compared to the current study, higher removal efficiency results were achieved, reaching 100% for the same amount of lignin incorporation. It is also important to highlight that the present study presents a relevant mechanical and swelling evaluation that is paramount when envisaging real environmental application that demands robust materials. For instance, previous results (Figure 4b) indicate that KL reduces swelling, which is vital to maintaining the membrane morphology and robustness that allows reuse.

### 4.3. Adsorption Mechanism

**Figure 7:**
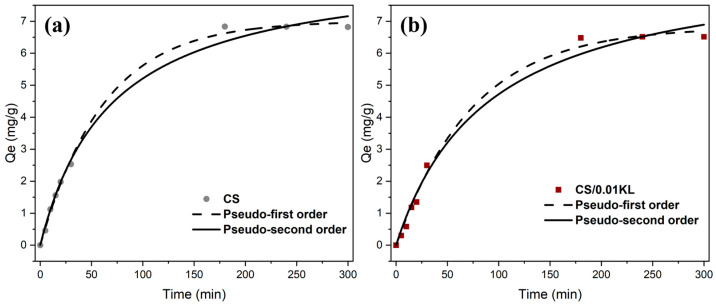
Fitting the pseudo-first-order and pseudo-second-order adsorption kinetics of the CS membrane (**a**) and the CS/0.01 KL membrane (**b**) ([Cr] initial = 5 mg/L; contact time: 5 h; pH = 7).

Studying the adsorption kinetics and adsorption mechanism is essential in terms of understanding the interaction between the absorbent and the contaminant. Thus, the data adsorption capacity (Qe, mg/g) vs. time (min) data were fitted to the pseudo-first [60] and pseudo-second-order kinetic [67] models. Figure 7a,b show both the model fitting of Cr(VI) adsorption kinetics on CS and CS/0.01 KL membranes. Table 3 shows the correlation coefficient (R^2^), estimated equilibrium capacity (Qe), and adsorption rate constants (k) for both models.

The correlation coefficients indicate that the pseudo-first and pseudo-second-order models are appropriate for describing the kinetic data, with R^2^ values exceeding 0.98. Regarding the CS membrane, the pseudo-first-order model is the most suitable for describing the Cr(VI) adsorption process, considering the R^2^ value (R^2^ = 0.998). This suggests that the adsorption is controlled by diffusion and mass transfer from the adsorbate to the adsorption site, which is physical adsorption or physisorption [89]. The adsorption of Cr(VI) using the lignin membrane (CS/0.01 KL) also fits the pseudo-first-order model (R^2^ = 0.98), indicating that the adsorption rate depends primarily on the availability of active sites on the surface of the CS/KL membranes. These active sites interact electrostatically with the protonated amino and hydroxyl groups of Cr(VI) metal ion removal [56]. Although the R^2^ of the pseudo-second-order model is close to the R2 of the pseudo-first-order model, the values of Qe obtained by the pseudo-second-order model are inconsistent with the experimental values, ruling out this model. One suggestion for the differences between the experimental (6.81 and 6.51 mg/g for the CS and CS/0.01 KL membranes, respectively) and theoretical (8.80 and 8.95 mg/g for the CS and CS/0.01 KL membranes, respectively) values of Qe from the pseudo-second-order model is a time lag, possibly due to a boundary layer or external resistance controlling at the beginning of the adsorption [90]. Although the results obtained are not in agreement with similar studies [91,92], suggesting that Cr(VI) adsorption occurs predominantly via chemical adsorption (following a pseudo-second-order model), we observed proximity in the R^2^ values of both models, indicating that the adsorption process may take place via physical and also chemical interactions.

**Figure 8:**
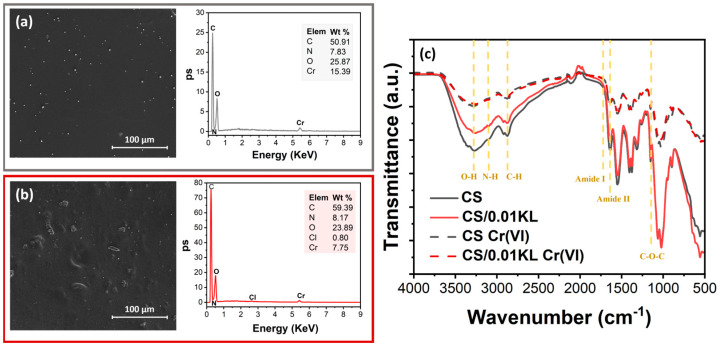
SEM images of the surface and respective SEM-EDX spectra after contact with Cr(VI): (**a**) CS and (**b**) CS/0.01 KL; (**c**) FTIR-ATR spectra of CS and CS/0.01 KL membranes before and after Cr(VI) adsorption.

After assessing the removal efficiency of the produced membranes, SEM-EDX (Figure 8a,b) analysis further confirmed the presence of the elements C, N, O, and Cr on their surface. The SEM-EDX spectrum of the CS/0.01 KL membrane, Figure 8b, shows one more element at a lower wt.% than the CS membrane: Cl. The absence of Cl in the CS membrane after Cr(VI) adsorption, Figure 8a, has already been reported in [93], which suggests that there is a ligand exchange reaction between the coordinated chlorate and the Cr(VI) ions. The adsorption of Cr(VI) ions by the composite was confirmed using the SEM images on both membranes and by the presence of the Cr peak in the EDS spectra, in which the CS membrane shows a more significant amount than the lignin membrane, 15.39 and 7.75 wt.%, respectively. 

FTIR-ATR analysis was carried out to investigate the mechanism of Cr(VI) adsorption on the membranes by comparing the spectra before and after Cr(VI) adsorption by the CS and CS/0.01 KL membranes (Figure 8c). It is observed that after contact with Cr(VI), no new bonds were visible in IR spectra. However, both membranes show a notable reduction in the intensity of the characteristic peaks of the functional groups (O-H and -NH bonds) of CS involved in the adsorption process, which can be related to chromium adsorption into the CS membrane. In a related study but using a composite of chitosan/ionic liquid to remove Cr(VI), there was a slight displacement and decrease in the intensity of the functional groups, indicating binding with the Cr(VI) ions [82]. Further, it has been suggested [86] that physical forces, specifically electrostatic interaction, between the chitosan composite and the Cr(VI) solution are responsible for the observed adsorption, which explains the absence of new bands in the FTIR-ATR spectra related to a new chemical bond between Cr and the CS membranes. Other studies using natural polymers for Cr(VI) removal have also shown similar results [94,95]. 

**Figure 9:**
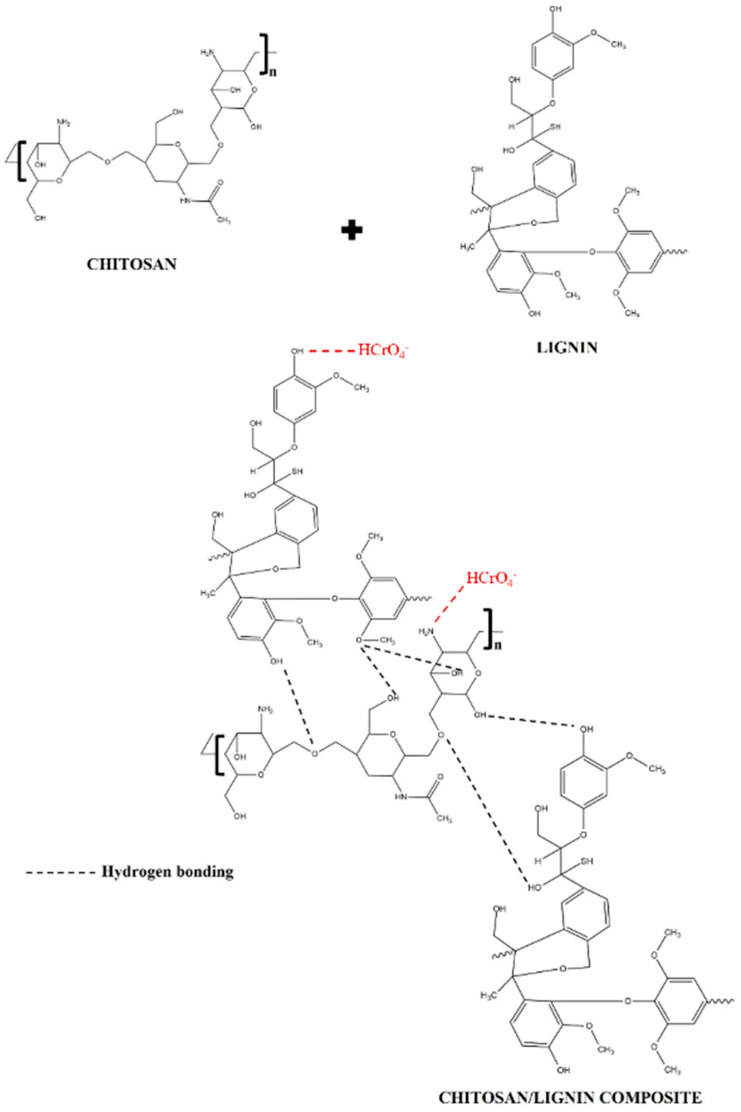
Possible interaction between chitosan and lignin and suggested mechanism for Cr(VI) adsorption by CS/KL membranes, adapted from [56,86].

A representation of the possible interactions of chitosan and lignin is shown in Figure 9. These interactions are probably due to the formation of weak hydrogen bonds between the functional groups of chitosan and lignin, which have already been suggested in [56]. Figure 9 also shows the possible Cr(VI) adsorption mechanism by CS/KL membranes. It indicates that the protonated amino and hydroxyl groups are sites of electrostatic interaction for the HCrO_4−_ ion. In other words, chromium is usually introduced as potassium dichromate, which gives a positive charge to the chromium atom in the solution [85]. This results in coordination bonds between the water molecules and the positively charged chromium ions.

It is important to note that it may be possible to recover the adsorbed chromium and reuse the composite membrane for multiple adsorption cycles. Since the mechanism is based on physical adsorption, changing the pH of the contact solution can promote Cr desorption and reactivation of the CS membranes.

## 5. Conclusions

Aquatic environment pollution is a growing problem, increasing the release of emerging contaminants into effluents, particularly heavy metals such as Cr(VI), which significantly negatively impact human health and the environment. Thus, this study developed CS and CS/KL membranes using a simple and low-cost method, solvent casting, at room temperature for Cr(VI) adsorption. It was found that the lignin did not introduce significant differences in the adsorption ability of the chitosan matrix. It provides mechanical reinforcement and reduces membrane swelling, aiding the material’s stability in aqueous environments. This is particularly relevant for the scope of water remediation applications. Studying the effect of varying solution pH showed that the CS/0.01 KL membrane presents higher Cr(VI) removal efficiency, 22% more than CS efficiency, at acidic or neutral pHs. The adsorption efficiency of the CS and CS/0.01 KL membranes after 300 min of contact showed excellent results of 100% removal of Cr(VI). SEM-EDX analysis confirmed the presence of chromium ions on the surface of the CS and CS/KL membranes after contact with the contaminated water. Future studies could focus on enhancing the regenerative capabilities of these membranes, exploring the adsorption efficiencies across a broader spectrum of contaminants, and the long-term stability and performance of these membranes in varied environmental conditions would be crucial to their practical deployment in water treatment facilities.

In conclusion, developing chitosan–lignin composite membranes represents a significant stride toward addressing the pressing challenge of heavy metal pollution in aquatic environments. By harnessing the synergistic properties of these bio-based polymers, this study lays the groundwork for advancing sustainable water remediation technologies that are both effective and environmentally benign. Future works will be mainly devoted to evaluating the reusability of the CS/KL membranes in Cr(VI) removal and studies in real water matrixes.

## Figures and Tables

**Table 1:** Tensile strength (σy), elongation at break (εb), and Young’s Modulus (E) of CS and CS with different KL content.

Membranes	Tensile Strength (σy) (MPa)	Elongation at Break (εb) (%)	Young’s Modulus (E) (MPa)
CS	55.58 ± 8.12	7.04 ± 2.02	15.95 ± 0.92
CS/0.01 KL	43.06 ± 5.75	8.30 ± 0.84	12.79 ± 1.54
CS/0.05 KL	47.78 ± 4.76	10.05 ± 3.54	13.49 ± 1.13

**Table 2:** Comparison of the Cr(VI) removal efficiency (%) of chitosan-based adsorbents.

Adsorbent	Adsorbent Dosage	Initial Concentration (mg/L)	Contact Time (min)	Removal Efficiency (%)	Reference
Chitosan-[BMIM][OAc]	1 g/L	100	360	<90	[82]
Chitosan grafted graphene oxide NP	2 g/L	50	420	96	[46]
Chitosan	0.5 g/L	10.686	300	52.51	[87]
Chitosan-EDPM (Ethylene propylene diene monomer)				53.36	
Chitosan/Lignin (10 mg)				91.69	
Chitosan/nanocellulose				92.33	
Chitosan-Silica-Hydroxyapatite	0.05 g	20–140	120	91	[88]
Chitosan	0.4 g	5/25/50	300	100	In this study
Chitosan/Lignin (0.01 g)				100	

**Table 3:** Parameters of pseudo-first-order and pseudo-second-order kinetics models for Cr(VI) adsorption by CS and CS/0.01 KL membranes.

	Membranes		
Models	Parameters	CS	CS/0.01 KL
Pseudo-First Order	K_1_ (min^−1^)	0.016	0.013
	Qe (mg/g) (exp)	7.005	6.817
	R^2^	0.998	0.994
Pseudo-Second Order	K_2_ (g·mg^−1^·min^−1^)	0.0016	0.0012
	Qe (mg/g) (exp)	8.803	8.954
	R^2^	0.994	0.988

## Data Availability

Data are contained within the article.
